# Changes in clinical features of adrenal Cushing syndrome: a national registry study

**DOI:** 10.1530/EC-24-0684

**Published:** 2025-05-12

**Authors:** Takuyuki Katabami, Shiko Asai, Ren Matsuba, Masakatsu Sone, Shoichiro Izawa, Takamasa Ichijo, Mika Tsuiki, Shintaro Okamura, Takanobu Yoshimoto, Michio Otsuki, Yoshiyu Takeda, Mitsuhide Naruse, Akiyo Tanabe, ACPA-J Study Group

**Affiliations:** ^1^ Department of Metabolism and Endocrinology, St. Marianna University Yokohama Seibu Hospital, Yokohama, Kanagawa, Japan; ^2^ Department of Metabolism and Endocrinology, St. Marianna University School of Medicine, Kawasaki, Kanagawa, Japan; ^3^ Division of Endocrinology and Metabolism, Tottori University Faculty of Medicine, Yonago, Tottori, Japan; ^4^ Department of Diabetes and Endocrinology, Saiseikai Yokohama-shi Tobu Hospital, Yokohama, Kanagawa, Japan; ^5^ Department of Endocrinology and Metabolism, National Hospital Organization Kyoto Medical Center, Kyoto, Japan; ^6^ Department of Endocrinology, Tenri Hospital, Nara, Japan; ^7^ Department of Diabetes and Endocrinology, Tokyo Metropolitan Hiroo Hospital, Tokyo, Japan; ^8^ Department of Molecular Endocrinology and Metabolism, Tokyo Medical and Dental University, Tokyo, Japan; ^9^ Department of Endocrinology, Tokyo Women’s Medical University, Tokyo, Japan; ^10^ Department of Metabolic Medicine, Osaka University Graduate School of Medicine, Osaka, Japan; ^11^ Department of Internal Medicine, Asanogawa General Hospital, Kanazawa, Ishikawa, Japan; ^12^ Endocrine Center, Ijinkai Takeda General Hospital, Kyoto, Japan; ^13^ Department of Diabetes, Endocrinology and Metabolism, National Center for Global Health and Medicine, Tokyo, Japan

**Keywords:** adrenal Cushing syndrome, clinical features, diagnosis, national registry, Japan

## Abstract

**Graphical abstract:**

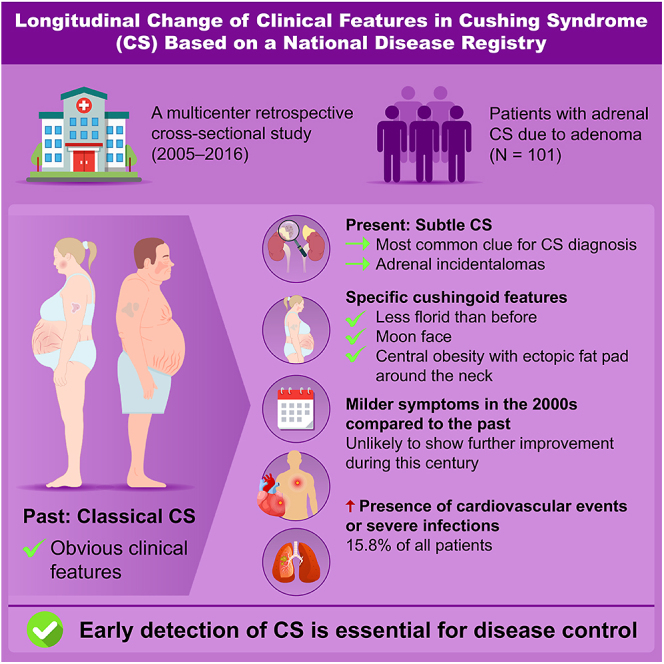

**Abstract:**

Adrenal Cushing syndrome (CS) has been rarely studied in recent years in Japan. This study aimed to investigate clinical characteristics and their changes over time in patients with adrenal CS. We analyzed 101 patients with adrenal CS caused by adenoma, dividing them into two groups based on diagnosis period: December 2011–November 2016 (later group, *n* = 50) and August 2005–November 2011 (earlier group, *n* = 51). Differences between the groups and comparisons with previous reports were assessed. Patients with subclinical CS were excluded. Adrenal incidentalomas were the most frequent reason for CS diagnosis (34%). Most patients exhibited few specific cushingoid features (2.5 ± 1.3), with moon faces and central obesity being the most common. Compared to earlier reports, specific cushingoid features were less frequent; nonetheless, no significant differences were observed between the earlier and later groups. All patients had midnight and post-dexamethasone suppression test serum cortisol levels exceeding 5 μg/dL. No significant differences were found between the groups regarding non-specific symptoms, endocrinological findings related to cortisol secretion, cardiometabolic commodities or infections, except for glucose intolerance and bone complications. The prevalence of metabolic disorders other than glucose intolerance and osteoporosis fluctuated over time. Sixteen patients developed cardiovascular diseases or severe infections. In conclusion, adrenal CS became less florid in the 2000s, showed no improvement in the following years, and remained associated with a high complication rate. Further research is needed to establish an early detection model for CS.

**Plain language summary:**

Our study found that one-sixth of patients with adrenal Cushing syndrome continued to develop severe complications in this century despite their specific cushingoid features being less pronounced than in the past. Notably, the findings provide clinical insights that may aid in earlier disease diagnosis.

## Introduction

Chronic exposure to excess glucocorticoids leads to Cushing syndrome (CS), with hypercortisolism causing a range of symptoms, signs and comorbidities, including arterial hypertension, diabetes mellitus, osteoporosis, severe infections and cardiovascular disease, all of which contribute to increased mortality (1, 2, 3, 4, 5). CS also negatively impacts quality of life and cognitive function, leading to worsening socioeconomic conditions; moreover, some of these effects persist even after remission (6, 7). Early diagnosis is therefore essential to reducing morbidity and mortality. A recent study (8) suggests that florid CS has become less common than previously reported, yet the time from symptom onset to diagnosis remains as long as 4 years (9, 10). A similar trend toward an increase in less florid CS is expected in Japan. However, to our knowledge, no nationwide epidemiological survey of adrenal CS has been conducted in Japan in recent decades.

The number of adrenal incidentalomas (AIs) detected through abdominal imaging has been increasing (11, 12), potentially aiding in the early diagnosis of adrenal CS. However, in most studies from other countries, adrenal CS accounts for a smaller proportion of all CS cases compared to Japan (20–47 vs >50%, respectively), despite a rise in incidence in recent reports (10, 13, 14, 15, 16). Consequently, there is limited evidence regarding diagnostic clues, clinical presentation, endocrinological findings and disease progression in a large cohort of patients with adrenal CS caused by adenomas in this century. This study aimed to examine the clinical phenotype, comorbidities and biochemical characteristics of Japanese patients with adrenal CS due to adenomas in the 2000s and to identify differences from previously reported findings.

## Materials and methods

### Study design and participants

This retrospective observational study was part of the Advancing Care and Pathogenesis of Intractable Adrenal Diseases in Japan (ACPA-J) study, which involved 10 referral centers (17, 18, 19). The ACPA-J was established to develop a disease registry and cohort for patients with subclinical adrenal CS, adrenal CS, primary macronodular adrenal hyperplasia or adrenocortical carcinoma. The study group collected clinical, biochemical, radiological and pathological data at enrollment to generate new evidence and inform clinical guidelines. Data were obtained from patients aged 20–90 years who were diagnosed with CS due to an adrenal adenoma between August 2005 and November 2016. The dataset used in this study were validated in March 2019. The study protocol was approved by the Ethics Committee of the National Center for Global Health and Medicine (Approval No.: NCGM-S-004259) and the ethics committees of the participating centers. This study adhered to the clinical research guidelines of the Ministry of Health, Labour and Welfare, Japan (MHLWJ) and the principles of the Declaration of Helsinki. Informed consent was obtained through an opt-out option available on the websites of each referral center.

**Figure 1:**
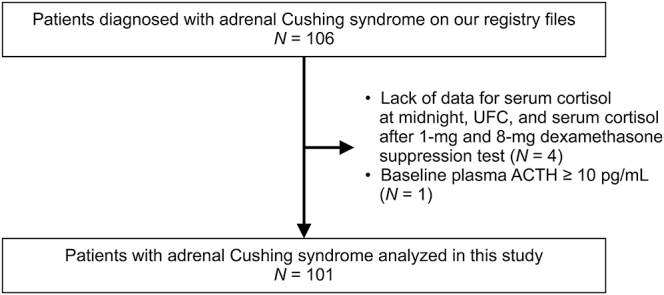
Flowchart of patient selection. ACTH, adrenocorticotropic hormone; UFC, urinary free cortisol.

In the ACPA-J study, adrenal diseases, including CS, were initially diagnosed by attending physicians. Patients with iatrogenic CS or CS caused by primary macronodular adrenal hyperplasia or adrenocortical carcinoma were excluded. Of the 106 patients diagnosed with adrenal CS due to adenomas, five were excluded for the following reasons: baseline plasma adrenocorticotropic hormone (ACTH) ≥10 pg/mL (*n* = 1) or significant missing data related to the hypothalamic-pituitary-adrenal axis (*n* = 4). None of the patients met the criteria for subclinical CS according to the Japan Endocrine Society clinical practice guidelines (20). Except for three cases, adrenal adenomas were pathologically confirmed through surgical specimens. In patients who did not undergo surgery, a tumor was classified as an adenoma if it appeared round or oval, hypodense (i.e., ≤10 Hounsfield units), homogeneous and well-defined on computed tomography (12). As a result, the final analysis included 101 patients with adrenal CS due to adrenal adenomas (Fig. 1).

The diagnosis of adrenal CS was validated based on the diagnostic criteria established by the Research on Intractable Diseases, Research Committee on Disorders of Adrenal Hormones from the MHLWJ in 2016 (21). These criteria included a combination of the following: the presence of specific and non-specific cushingoid features, confirmation of cortisol hypersecretion through elevated morning serum cortisol levels (generally ≥20 μg/dL) and/or high 24 h urinary free cortisol (UFC; typically more than four times the upper limit of normal (ULN) for the assay used at each center), disruption of the circadian rhythm in serum cortisol levels (serum cortisol at 21:00–23:00 h ≥5 μg/dL), suppression of ACTH secretion (morning plasma ACTH <10 pg/mL and/or a blunted response to corticotropin-releasing hormone (CRH) stimulation, defined as either an increase of <1.5 times the baseline ACTH or peak ACTH <10 pg/mL), failure to suppress serum cortisol levels (≥5 μg/dL) after the standard overnight 1 mg and/or 8 mg dexamethasone suppression test (DST), and the presence of an adrenal tumor on imaging.

### Measurements

The collected data included patient demographics such as age at diagnosis, sex, body mass index (BMI) and the reason for diagnosing CS. Specific cushingoid features recorded were moon face, dorsocervical or subclavian fat pad, central obesity, easy bruising, thin skin, muscle weakness, purple striae and facial plethora. Non-specific cushingoid features included acne, virilism or hirsutism in women, psychiatric disorders, menstrual irregularity and leg edema. Biochemical and hormonal profiles were assessed, including hemoglobin A1c (HbA1c), low-density lipoprotein cholesterol (LDL-C), high-density lipoprotein cholesterol (HDL-C), triglycerides (TG), morning and midnight serum cortisol, serum cortisol after the 1 mg or 8 mg DST, plasma ACTH before and after CRH stimulation, 24 h UFC and plasma dehydroepiandrosterone sulfate (DHEA-S). Comorbidities examined included hypertension, impaired glucose tolerance, dyslipidemia, obesity, bone fracture, osteoporosis, venous thromboembolism, cerebral infarction, cerebral hemorrhage, angina pectoris, myocardial infarction, heart failure, pneumonia, sepsis, deep abscess and other infections. Adrenal tumor diameter was assessed using imaging. To systematically assess various measurements, including specific and non-specific cushingoid features in patients with adrenal CS, we predefined survey items before initiating the study. We did not predefine the period for the major adverse cardiovascular and cerebrovascular events (MACCEs) and serious infections. The diseases were registered only if attending physicians determined they were associated with hypercortisolism. Missing data were excluded from the analysis. UFC and serum cortisol levels were partially expressed as multiples of the ULN or lower limit of normal (LLN) due to changes in assay methods. Further details on assay methods are provided in the supplementary data (see section on Supplementary materials given at the end of the article).

Hypertension was defined as a blood pressure of ≥140/90 mmHg or the use of antihypertensive medication (22). Due to inconsistencies in registration data, prediabetes and type 2 diabetes have been classified together under impaired glucose tolerance. Impaired glucose tolerance was defined as a fasting plasma glucose level of ≥110 mg/dL, a 2 h plasma glucose level of ≥140 mg/dL after a 75 g oral glucose load, an HbA1c level of ≥6.2% or current antidiabetic therapy (23). Dyslipidemia was defined by LDL-C levels ≥140 mg/dL, HDL-C levels <40 mg/dL, TG levels ≥150 mg/dL or the use of lipid-lowering therapy (24). Obesity was classified as a BMI ≥25 kg/m^2^, following the criteria of the Japan Society for the Study of Obesity (25). Osteoporosis was diagnosed based on a T-score ≤−2.5 standard deviation (SD) on dual-energy X-ray absorptiometry, in accordance with World Health Organization criteria (26). The presence of other symptoms, signs or comorbidities beyond the listed conditions was determined by the attending physicians based on medical records. The prevalence of MACCEs was also calculated. The CRH loading test is used to assess ACTH suppression in patients with suspected ACTH-independent hypercortisolism (20). A normal ACTH response to CRH stimulation was defined as plasma ACTH levels exceeding 10 pg/mL and increasing by more than 50% from baseline.

### Classification of participants according to the date of diagnosis

The primary objective of this study was to examine temporal changes in the clinical presentation of adrenal CS, necessitating classification based on the date of diagnosis. We also sought to clarify recent trends in CS diagnosis. The most recent diagnosis among study participants was recorded in November 2016. To analyze changes in clinical presentation over 10 years, we classified patients into two groups: those diagnosed within 5 years of the most recent case (i.e., December 2011–November 2016, later group; *n* = 50) or those diagnosed earlier (i.e., August 2005–November 2011, earlier group; *n* = 51).

### Changes in the clinical pictures over time

To examine changes in the clinical picture over time, we compared the prevalence of symptoms, signs and comorbidities in this study with findings from a nationwide survey conducted by the Research on Intractable Diseases, Research Committee on Disorders of Adrenal Hormones under the MHLWJ in 1997 (16) and data from traditional reports compiled by Rosset *et al.* (8). The nationwide survey was conducted in 1997 and 1998 using questionnaires sent to 4,060 departments. It included 737 patients with CS, covering adrenal CS caused by adenoma and bilateral hyperplasia, pituitary CS and ectopic ACTH syndrome, with adrenal CS accounting for 47.1% of cases. While the later research did not provide details on patient numbers, study duration or data collection methods, the data sources were clearly stated.

### Statistical analysis

Statistical analyses were conducted using SPSS (version 26.0; IBM Corp., USA) or EZR (Saitama Medical Center, Jichi Medical University, Japan) (27). Results are expressed as means ± SDs and frequencies (positive/total observations) unless otherwise specified. Data distributions were assessed using the Kolmogorov–Smirnov test. Quantitative variables were compared between groups using the Student’s *t*-test, while the categorical variables were analyzed using the *χ*^2^ test or Fisher’s exact test. We used a single-sample binomial test to compare our variable frequencies with those in previous studies (8). Statistical significance was defined as a *P*-value of <0.05.

## Results

### Clinical characteristics

This study included 101 patients with adrenal CS, with a higher prevalence in women than men. The average age of participants was 46.9 ± 13.3 years, with only 20% aged over 60 (Table 1). Notably, AIs were the most frequent finding leading to a CS diagnosis, followed by hypertension. Specific cushingoid features, such as moon face and muscle weakness, prompted diagnosis in approximately 15% of cases. The mean maximum diameter of the adenomas was approximately 3 cm. More than 90% of patients (94/101) had adrenal adenomas >2 cm. Bilateral adenomas were observed in nearly 20% of the study population. No significant differences were observed between the earlier and later groups regarding age, sex distribution, diagnostic triggers (except fractures), adenoma size or the prevalence of bilateral adenomas.

**Table 1:** Clinical characteristics of patients with Cushing syndrome.

	All patients with Cushing syndrome	Earlier group	Later group	*P*-value
	*n* = 101	*n* = 51	*n* = 50	
Age, years	46.9 (13.3)	45.9 (13.3)	47.8 (13.4)	0.459
20–39/40–59/>60, *n* (%)	30/50/20 (30.0%/50.0%/20.0%)	19/21/10 (38.0%/42.0%/20.0%)	11/29/10 (22.0%/58.0%/20.0%)	0.181
Female, *n* (%)	90/100 (90.0%)	45/50 (90.0%)	45/50 (90.0%)	0.999
BMI, kg/m^2^	24.6 (4.3)	24.9 (4.3)	24.4 (4.2)	0.545
Reasons leading to Cushing syndrome diagnosis				
Incidentaloma, *n* (%)	34/101 (33.7%)	17/51 (33.3%)	17/50 (34.0%)	0.999
Hypertension, *n* (%)	30/101 (29.7%)	16/51 (31.4%)	14/50 (28.0%)	0.828
Moon face, *n* (%)	11/101 (10.9%)	8/51 (15.7%)	3/50 (6.0%)	0.2
Weight gain, *n* (%)	10/101 (9.9%)	4/51 (7.8%)	6/50 (12.0%)	0.525
Edema, *n* (%)	10/101 (9.9%)	5/51 (9.8%)	5/50 (10.0%)	0.999
Fracture, *n* (%)	8/101 (7.9%)	1/51 (2.0%)	7/50 (14.0%)	0.031
Muscle weakness, *n* (%)	4/101 (4.0%)	3/51 (5.9%)	1/50 (2.0%)	0.617
Bilateral adrenal tumors, *n* (%)	17/101 (16.8%)	11/51 (21.6%)	6/50 (12.0%)	0.308
Maximum diameter of tumor (mm)	28.4 (7.6)	27.2 (7.2)	29.6 (7.9)	0.111
≥20 mm, *n* (%)	94 (94.0%)	47 (92.2%)	47 (95.9%)	0.678

Data are presented as mean (SD) or number of patients (%). Patients were categorized into two groups based on their diagnosis date: within 5 years of the most recent case (December 2011–November 2016, later group) or earlier (August 2005–November 2011, earlier group).

*P*-values were calculated using Student’s *t*-test. Proportions between the before and after groups were compared using the *X*^2^ or Fisher’s exact tests.

BMI, body mass index.

### Specific and non-specific cushingoid features

Most patients with CS exhibited a limited number of specific features (mean ± SD, 2.5 ± 1.3) (Table 2). Nearly 40% of patients had two or fewer specific cushingoid features, while only 5% had five or more. The most frequently observed feature was moon face, followed by central obesity with a dorsocervical or subclavian fat pad, easy bruising or thin skin, facial plethora and muscle weakness or purple striae. The two most common features were present in over 50% of patients. Non-specific cushingoid features, including menstrual irregularity, acne, psychiatric disorders, hirsutism, virilization in women and edema, were observed in fewer than 25% of cases. The mean number of non-specific features was approximately one (0.6 ± 0.7). No significant differences in symptoms and signs of CS were found between the earlier and later groups.

**Table 2:** Presence of specific and non-specific cushingoid features.

	All patients with Cushing syndrome	Earlier group	Later group	*P*-value
Cushingoid appearance, *n* (%)	99/101 (98.0%)	51/51 (100%)	48/50 (96.0%)	0.243
Specific features				
(1) moon face, *n* (%)	85/101 (84.2%)	41/51 (80.4%)	44/50 (88.0%)	0.439
(2) central obesity, *n* (%)	60/101 (59.4%)	32/51 (62.7%)	28/50 (56.0%)	0.626
(3) easy bruising or thin skin, *n* (%)	45/101 (44.6%)	19/51 (37.3%)	26/50 (52.0%)	0.163
(4) facial plethora, *n* (%)	25/101 (24.8%)	10/51 (19.6%)	15/50 (30.0%)	0.327
(5) muscle weakness, *n* (%)	21/101 (20.8%)	10/51 (19.6%)	11/50 (22.0%)	0.959
(6) purple striae, *n* (%)	21/101 (20.8%)	14/51 (27.5%)	7/50 (14.0%)	0.156
Non-specific features				
(7) menstrual irregularity, *n* (%)	20/79 (25.3%)	10/37 (27.0%)	10/42 (23.8%)	0.945
(8) acne, *n* (%)	15/101 (14.9%)	8/51 (15.7%)	7/50 (14.0%)	0.999
(9) psychiatric disorders, *n* (%)	13/101 (12.9%)	7/51 (13.7%)	6/50 (12.0%)	0.999
(10) hirsutism or virilization in female, *n* (%)	9/85 (10.6%)	6/41 (14.6%)	3/44 (6.8%)	0.303
(11) leg edema, *n* (%)	4/101 (4.0%)	4/51 (7.8%)	0/50 (0.0%)	0.118
Number of items				
In specific features ((1)–(6)), mean (SD)	2.5 (1.3)	2.5 (1.2)	2.6 (1.4)	0.562
In non-specific features ((7)–(11)), mean (SD)	0.6 (0.7)	0.7 (0.8)	0.5 (0.7)	0.258

Data are presented as mean (SD) or number of patients (frequency). Patients were categorized into two groups based on their diagnosis date: within 5 years of the most recent case (December 2011–November 2016, later group) or earlier (August 2005–November 2011, earlier group).

*P*-values were calculated using Student’s *t*-test. Proportions between the before and after groups were compared using the *X*^2^ or Fisher’s exact tests.

### Endocrinological findings

**Figure 2:**
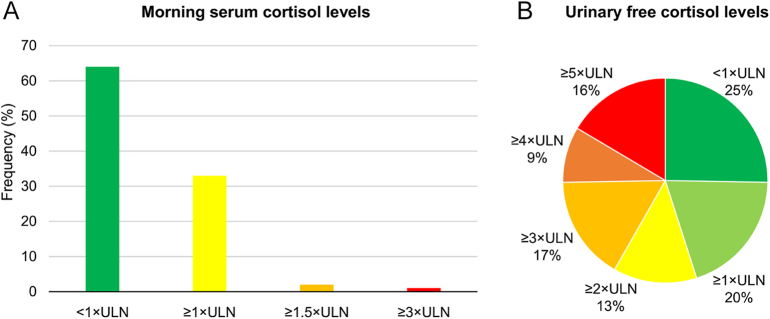
Distribution of the ratio of morning serum (left) cortisol and (right) urinary free cortisol levels to the upper limit of normal (ULN).

Serum cortisol levels after the 1 mg or 8 mg DST and midnight serum cortisol levels exceeded 5.0 μg/dL in all participants who underwent these tests (Table 3). In addition, all patients had markedly low baseline plasma ACTH levels. More than 50% of patients had morning serum cortisol levels below the ULN, while over 25% had UFC levels below this threshold (Fig. 2). Absolute serum cortisol concentrations (μg/dL) following the 8 mg DST were higher in the earlier group than in the latter group. However, when expressed as multiples of the LLN, there was no difference between groups, suggesting that this discrepancy was due to variations in assay methods. In contrast, baseline plasma ACTH levels were higher in the earlier group than in the latter group. Other parameters related to the hypothalamic-pituitary-adrenal axis, such as morning, midnight and post-DST serum cortisol levels, UFC levels, serum DHEA-S levels and plasma ACTH levels after CRH stimulation, were comparable between groups. The CRH stimulation test was performed in about 33% of participants. All but one patient had peak plasma ACTH levels below 10 pg/mL after CRH loading.

**Table 3:** Endocrinological findings.

		All patients with Cushing syndrome	Earlier group	Later group	*P*-value
		*n* = 101	*n* = 51	*n* = 50	
Morning serum cortisol levels (*n* = 100)	μg/dL	17.7 (5.7)	18.4 (4.8)	17.0 (6.5)	0.232
× the ULN	times	0.90 (0.3)	0.96 (0.3)	0.88 (0.4)	0.264
Midnight serum cortisol levels (*n* = 97)	μg/dL	17.6 (5.3)	18.6 (4.7)	16.7 (5.8)	0.088
≥5 μg/dL	*n* (%)	97/97 (100%)	48/48 (100%)	49/49 (100%)	N/A
× the lower limit of normal	times	3.2 (1.3)	3.2 (1.3)	3.2 (1.3)	0.846
Plasma ACTH levels in the morning (*n* = 100)	pg/mL	1.9 (1.7)	2.6 (2.0)	1.2 (0.9)	<0.001
<10 pg/mL	*n* (%)	100/100 (100%)	50/50 (100%)	50/50 (100%)	N/A
DHEA-S (*n* = 97)	μg/dL	40.7 (50.6)	35.2 (34.3)	45.8 (61.8)	0.313
Urinary free cortisol (*n* = 91)	mg/24 h	283.1 (329.8)	279.8 (273.2)	285.8 (372.5)	0.932
× the ULN	times	3.5 (4.1)	3.5 (3.4)	3.6 (4.6)	0.928
Serum cortisol levels after 1 mg DST (*n* = 96)	μg/dL	18.6 (5.4)	19.3 (4.4)	17.9 (6.2)	0.202
≥5 μg/dL	*n* (%)	96/96 (100%)	48/48 (100%)	48/48 (100%)	N/A
× the LLN	times	3.4 (1.4)	3.3 (1.3)	3.5 (1.4)	0.566
Serum cortisol levels after 8 mg DST (*n* = 71)	μg/dL	18.6 (5.2)	19.9 (5.2)	17.0 (5.0)	0.017
≥5 μg/dL	*n* (%)	71/71 (100%)	38/38 (100%)	33/33 (100%)	N/A
× the LLN	times	3.4 (1.3)	3.5 (1.5)	3.4 (1.2)	0.775
Peak plasma ACTH value after CRH stimulation test (*n* = 36)	pg/mL	3.4 (3.4)	3.9 (1.5)	2.9 (4.3)	0.413

Data are presented as mean (SD) or number of patients (%). Patients were categorized into two groups based on their diagnosis date: within 5 years of the most recent case (Dec 2011–Nov 2016, later group) or earlier (Aug 2005–Nov 2011, earlier group).

*P*-values were calculated using Student’s *t*-test. Proportions between the before and after groups were compared using the *X*^2^ or Fisher’s exact tests.

ACTH, adrenocorticotropic hormone; CRH, corticotropin-releasing hormone; DHEA-S, dehydroepiandrosterone sulfate; DST, dexamethasone suppression test; N/A, not available; LLN, lower limit of normal; ULN, upper limit of normal.

### Comorbidities

Among cardiometabolic conditions, hypertension was the most prevalent comorbidity (79.2%), followed by dyslipidemia, bone disorders, obesity and glucose intolerance (Table 4). The incidence of venous thromboembolism was 4.2%. Apart from all fractures or osteoporosis, no significant differences in complication rates were observed between the groups. Table 5 presents the frequency of MACCEs and severe infections among participants. Thirteen MACCEs (10.9%), including cerebral infarction or hemorrhage, angina pectoris, myocardial infarction and heart failure, were reported in 11 patients. In addition, six patients (6.0%) developed severe infections, such as pneumonia, sepsis or deep abscesses. Overall, 16 (15.8%) patients experienced serious illnesses. The prevalence of these conditions did not differ significantly between the earlier and later groups.

**Table 4:** Comorbidities in patients with Cushing syndrome.

	All patients with Cushing syndrome	Earlier group	Later group	*P*-value
	*n* = 101	*n* = 51	*n* = 50	
Cardiometabolic				
Hypertension, *n* (%)	80/101 (79.2%)	42/51 (82.4%)	38/50 (76.0%)	0.588
Dyslipidemia, *n* (%)	61/99 (61.6%)	32/50 (64.0%)	29/49 (59.2%)	0.775
Obesity (BMI ≥25 kg/m^2^), *n* (%)	39/96 (40.6%)	23/48 (47.9%)	16/48 (33.3%)	0.212
Impaired glucose tolerance, *n* (%)	33/101 (32.7%)	17/51 (33.3%)	16/50 (32.0%)	1
Bone				
All fractures, *n* (%)	25/93 (26.9%)	9/45 (20.0%)	16/48 (33.3%)	0.224
Osteoporosis, *n* (%)	42/90 (46.7%)	17/42 (40.5%)	25/48 (52.1%)	0.374
All fractures or osteoporosis, *n* (%)	48/101 (47.5%)	18/51 (35.3%)	30/50 (60.0%)	0.017
Coagulopathy				
Venous thromboembolism, *n* (%)	4/96 (4.2%)	3/50 (6.0%)	1/46 (2.2%)	0.670

Patients were categorized into two groups based on their diagnosis date: within 5 years of the most recent case (December 2011–November 2016, later group) or earlier (August 2005–November 2011, earlier group). BMI, body mass index.

**Table 5:** Number of cardiovascular disease and infection events.

	All patients with Cushing syndrome	Earlier group	Later group	*P*-value
	*n* = 101	*n* = 51	*n* = 50	
MACCEs, *n* (%)	11/101 (10.9%)	6/51 (11.8%)	5/50 (10%)	1
Cerebral infarction, *n* (%)	2/101 (2.0%)	1/51 (2.0%)	1/50 (2.0%)	1
Cerebral hemorrhage, *n* (%)	0/101 (0%)	0/51 (0%)	0/50 (0%)	N/A
Angina pectoris, *n* (%)	2/101 (2.0%)	2/51 (3.9%)	0/50 (0%)	0.484
Myocardial infarction, *n* (%)	2/101 (2.0%)	1/51 (2.0%)	1/50 (2.0%)	1
Heart failure, *n* (%)	7/101 (6.9%)	4/51 (7.8%)	3/50 (6.0%)	1
Severe infection, *n* (%)	6/101 (6.0%)	4/51 (7.8%)	2/50 (4.1%)	0.678
Pneumonia, *n* (%)	2/101 (2.0%)	1/51 (2.0%)	1/50 (2.0%)	1
Deep abscess, *n* (%)	2/101 (2.0%)	1/51 (2.0%)	1/50 (2.0%)	1
Sepsis, *n* (%)	1/101 (1.0%)	1/51 (2.0%)	0/50 (0%)	1
Other infections, *n* (%)	1/101 (1.0%)	1/51 (2.0%)	0/50 (0%)	1

Patients were categorized into two groups based on their diagnosis date: within 5 years of the most recent case (December 2011–November 2016, later group) or earlier (August 2005–November 2011, earlier group). MACCEs, major adverse cardiovascular and cerebrovascular events; N/A, not available.

### Changes in the clinical presentation over time

To assess temporal changes in the clinical presentation, we compared the prevalence of symptoms, signs and comorbidities in this study with data from a nationwide survey conducted by the MHLWJ in 1997 (16) and traditional reports compiled by Rosset *et al.* (8) (Supplementary Table 1). The frequency of specific cushingoid features, except for moon face, and non-specific cushingoid features, such as diabetes mellitus, menstrual irregularities, obesity and dyslipidemia, was significantly lower in our cohort compared with previous reports. The trends in hypertension, depression and osteoporosis varied by region. In addition, significant differences in the prevalence of easy bruising, hypertension and osteoporosis were observed between the earlier and later groups.

## Discussion

This multicenter study in Japan demonstrated that fully developed adrenal CS has been identified less frequently in the twenty-first century compared with the previous century, and clinical outcomes did not improve during the 2000s. One possible reason for the increased detection of less florid CS is the higher likelihood of encountering AIs, as AIs discovery led to CS diagnosis in approximately 33% of the study cohort. Similar trends have been observed in West and North Africa (10, 14, 15, 16). In addition, Braun *et al.* (28) reported that the presence of AIs independently increased the likelihood of a CS diagnosis. However, the incidence of AIs far exceeds that of CS (11, 12). Given that the Endocrine Society’s practice guidelines for CS (29) advise against widespread testing for all suspected cases, additional information is needed to enhance the pretest probability for detecting CS. In this study, only one patient (1/100, 1%) was male with an adrenal tumor smaller than 2.0 cm (7/101, 6.0%), suggesting that clinical evaluation can significantly reduce the likelihood of CS.

To assess the impact of AIs on early CS detection, we categorized adrenal CS patients into two groups based on whether their diagnosis resulted from AIs (*n* = 34) or not (*n* = 67). The mean number of specific cushingoid features was comparable between the two groups (2.3 ± 1.4 vs 2.7 ± 1.2, *P* = 0.119, data not shown). Similar trends were observed in non-specific cushingoid features, endocrinological findings, comorbidities and MAACEs. Conversely, when categorized based on having fewer than two specific cushingoid features (*n* = 21) versus two or more (*n* = 80), the detection rate of AIs tended to be higher, and serum cortisol levels at midnight or after a 1 mg DST were lower in those with fewer features than in those with more pronounced features (52.4 vs 28.7%, *P* = 0.067; 15.4 ± 4.4 μg/dL vs 18.2 ± 5.4 μg/dL, *P* = 0.031; and 16.3 ± 5.0 μg/dL vs 19.1 ± 5.3 μg/dL, *P* = 0.03, respectively, data not shown). Furthermore, the Cochran–Armitage test indicated that the trend across the diagnosis rate of CS leading to AIs rose with an increasing number of positive findings of specific cushingoid features (*P* = 0.035, data not shown). These findings suggest that while AIs may aid in identifying patients with less florid CS, they are unlikely to contribute to earlier diagnosis.

Cushingoid features can be categorized as specific or non-specific. Specific features help differentiate patients with severe CS from those without CS or those with cardiometabolic disorders or AIs with mild autonomous cortisol secretion (30). In this study, a moon face was observed in over 80% of participants, making it the most prevalent specific cushingoid feature. This suggests that a moon face may appear early and/or serve as the first distinct sign in most CS cases. Therefore, when evaluating patients at risk for CS, physicians should compare past and current photographs to facilitate early diagnosis. The development of advanced facial recognition software capable of detecting facial changes over time could further aid in preventing missed diagnoses of CS (31, 32). In addition, central obesity, defined by a dorsocervical and/or subclavian fat pad, was present in over 50% of CS cases, whereas obesity based on BMI criteria was observed in approximately 40% (24). The rising global prevalence of overweight and obesity complicates the diagnosis of CS. However, general obesity may negatively impact CS prediction (33). Our findings suggest that body shape, fat distribution – including the presence of a distinct fad pad – and facial contour are more relevant than body weight in distinguishing CS from general obesity. This distinction may help reduce unnecessary testing for CS.

Consistent with previous studies (33, 34), cardiometabolic conditions such as metabolic syndrome and bone comorbidities (i.e., osteoporosis and fractures) were frequently observed in patients with CS. However, as noted earlier, the prevalence of AIs with mild cortisol hypersecretion is significantly higher than that of CS, and non-specific cortisol-related cardiometabolic comorbidities are also common in AIs (34). Because these conditions are prevalent in the general population, broad screening has not been endorsed, as some non-specific features (e.g., hypertension, obesity and glucose intolerance) are more likely to indicate non-CS (35). Therefore, as recommended by clinical guidelines (29), additional factors – such as comorbidities that develop atypically with age, worsen over time or appear sequentially – should be considered before initiating screening. Moreover, in this study, 19 MACCEs or severe infections requiring hospitalization were reported in 16 patients (15.8%). This underscores the fact that, even in the 2000s, delays in diagnosing adrenal CS persist, necessitating improvements to reduce complications. Similarly, Rubinstein *et al.* (10) found no evidence of earlier CS diagnosis in patients treated after 2000 compared to studies conducted before 2000.

Our study revealed four notable findings in the endocrinological data. First, we confirm that CS should not be ruled out even if morning serum cortisol levels are normal, as this was observed in 66% of our patients. Endocrinologists must inform general practitioners to prevent missed diagnoses of CS. Second, post-1 mg DST serum cortisol levels in our cohort were much higher than the 1.8 μg/dL (50 nmol/L) cutoff recommended by the Endocrine Society Practical Guideline (29), consistently exceeding 5.0 μg/dL (138 nmol/L). Ceccato *et al.* (33) suggested a new threshold of 7.1 μg/dL (196 nmol/L) to distinguish CS from AIs without CS and 2.4 μg/dL (66 nmol/L) to differentiate CS from non-CS. We considered adjusting DST cutoffs based on the patient’s circumstances (e.g., the presence or absence of AIs or specific cushingoid features). Recent guidelines state that cortisol autonomy exists on a biological continuum, without a distinct separation between nonfunctioning and functioning adenomas with varying degrees of cortisol excess (12). Any post-DST cortisol cutoff value generally demonstrates poor accuracy in predicting prevalent comorbidities in patients with AIs. However, this finding applies to patients without overt CS, as the risk of developing CS is very low in the absence of clinical signs at the initial assessment. Furthermore, adrenal adenomas associated with overt CS have shown a distinct mutation profile compared to those with mild autonomous cortisol secretion (36). These results suggest that the two types of adenomas should be distinguished. Our data indicate that if serum cortisol levels after DST are significantly higher than the current cutoff value (i.e., 1.8 μg/dL), physicians should carefully assess patients for specific cushingoid features. A large-scale nationwide study in Japan, including adrenal CS, AIs with autonomous cortisol secretion, and non-CS, is needed to determine the optimal serum cortisol level cutoff after a DST for diagnosing adrenal CS in the Japanese population.

Third, normal UFC levels were found in 25% of participants despite elevated serum cortisol levels after the DST or at midnight in all patients. Several factors such as urinary volume, adherence to proper urine collection, day-to-day variability, and the number of measurements can affect UFC levels (37). To assess the impact of renal function on these results, we analyzed the estimated glomerular filtration rate (eGFR) in patients with normal UFC levels. The mean UFC levels were lower in patients with an eGFR <60 mL/min/m^2^ (*n* = 22) than in those with an eGFR ≥60 mL/min/m^2^ (*n* = 68) (1.0 ± 0.8 × ULN vs 4.0 ± 4.3 × ULN, *P* = 0.016), suggesting that renal impairment partially contributed to the discrepancies. Unfortunately, other factors affecting the results were not available in our data. Finally, all but one patient (97.3%) had peak plasma ACTH levels <10 pg/mL after CRH stimulation. This test may yield pseudo-positive results, as the exceptional patient had five specific cushingoid features along with typical autonomous cortisol secretion in CS (e.g., serum cortisol levels at midnight and after 1 mg DST near 20 μg/dL). Thus, the CRH stimulation test may not provide additional information for most patients with adrenal CS exhibiting clear ACTH suppression.

This study has several limitations, primarily due to its retrospective, cross-sectional design. First, selection bias may have occurred due to differences in data handling across participating centers, endocrine tests related to CS, or assay methods for CS-related comorbidities. Second, there were varying numbers of patients available for each measurement. Third, the absence of a predefined diagnostic protocol for CS and its comorbidities may have contributed to inconsistencies in diagnosis. Fourth, comparisons were challenging due to the wide variability in assay methods. Fifth, a 5-year period may be insufficient to evaluate changes in the clinical presentation of CS over time. Finally, as the study was conducted solely in Japan and primarily referenced Japanese CS and/or subclinical CS clinical guidelines (20, 21), its findings may not be generalizable. However, a key strength of this study is its involvement of multiple centers and a larger sample size compared to previous studies.

In conclusion, cases of adrenal CS in the 2000s were less florid than in previous decades although no further clinical improvement was observed during this century. A new model for the early detection of CS is necessary, as the prevalence of CS-related complications remains high. To reduce the time to diagnosis of adrenal CS, it is important to avoid overlooking moon face and central obesity with dorsocervical and/or subclavian fat pad, assess morning ACTH and serum cortisol after a DST with higher cutoff values than those recommended by the Endocrine Society, use abdominal computed tomography, and consider tumor size and patient sex when evaluating patients with suspected CS. Additional studies are needed to create a more effective diagnostic method for earlier identification of CS.

## Supplementary materials



## Declaration of interest

The authors declare that there are no conflicts of interest that could be perceived as affecting the impartiality of the research presented.

## Funding

This research was supported by the National Center for Global Health and Medicinehttps://doi.org/10.13039/100012319, Japan (grant numbers 21A1015, 24A1004), the MHLWJ (grant number Nanbyo-Ippan-23FC1041) and AMED, Japanhttps://doi.org/10.13039/100009619 (grant numbers JP17ek010922, JP20ek0109352).

## Author contribution statement

Takuyuki Katabami (conceptualization (lead), methodology (lead), validation (equal), visualization (lead), writing–original draft (lead), writing–review and editing (equal)), Shiko Asai (data curation (lead), formal analysis (lead), investigation (equal), software (equal), visualization (equal), writing–review and editing (equal)), Ren Matsuba (data curation (equal), formal analysis (lead), investigation (equal), software (equal), visualization (equal), writing–review and editing (equal)), Masakatsu Sone (data curation (equal), investigation (supporting), writing–review and editing (supporting)), Shoichiro Izawa (data curation (equal), investigation (supporting), writing–review and editing (supporting)), Takamasa Ichijo (data curation (equal), investigation (supporting), writing–review and editing (supporting)), Mika Tsuiki (data curation (equal), investigation (supporting), writing–review and editing (supporting)), Shintaro Okamura (data curation (equal), investigation (supporting), writing–review and editing (supporting)), Takanobu Yoshimoto (data curation (equal), investigation (supporting), writing–review and editing (supporting)), Michio Otsuki (data curation (equal), investigation (supporting), writing–review and editing (supporting)), Yoshiyu Takeda (data curation (equal), investigation (supporting), writing–review and editing (supporting)), Mitsuhide Naruse (data curation (equal), project administration (equal), supervision (lead), validation (lead), writing–review and editing (lead)), Akiyo Tanabe (data curation (equal), funding acquisition (lead), project administration (equal), resource (lead), supervision (lead), validation (lead), writing–review and editing (lead)), ACPA-J Study Group (data curation (equal), investigation (supporting), writing–review and editing (supporting)).

## Data availability

The data supporting this article cannot be shared publicly due to restrictions imposed by the authors’ institutes. Data can be made available upon reasonable request to the corresponding author.
