# Supplementary material for: Changes in clinical features of adrenal Cushing syndrome: a national registry study
Source: Endocr Connect. 2025 May 12;14(5):e240684. doi: 10.1530/EC-24-0684 (PMC12070466; doi:10.1530/EC-24-0684)
Supplement: Supplementary file 1 [file supplementary_materials.pdf]

Supplementary Data

Assay methods

Serum cortisol concentrations were measured using an electrochemiluminescence immunoassay kit (Elecsys cortisol II; Roche Diagnostics, Tokyo, Japan) at two centers, a chemiluminescent immunoassay kit (Chemilumi cortisol II; Siemens Healthineers, Tokyo, Japan) at two centers, a chemiluminescent immunoassay kit (Immulite 2000 cortisol; Siemens Healthineers) at one center, an enzyme immunoassay kit (E-test TOSOH II; TOSOH, Tokyo, Japan) at three centers, and a chemiluminescent immunoassay kit (Immulite 2000 cortisol; Beckman Coulter, Tokyo, Japan) at two centers. The reference ranges of these kits were 7.2–63.3, 7.0–56.0, and <46 pg/mL, respectively. Urinary free cortisol levels were measured using a radioimmunoassay kit (Cortisol kit FR; Fuji Rebio Co. Ltd., Tokyo, Japan) at nine centers, and a chemiluminescent immunoassay kit (Chemilumi cortisol II; Siemens Healthineers) at one center, with reference ranges of 11.2–80.3 and 26.0–187.0 µg/24-h, respectively. Plasma ACTH concentrations were determined using an electrochemiluminescence immunoassay kit (Elecsys; Roche Diagnostics) at seven centers, an immunoradiometric assay kit (IRMA; Mitsubishi Chemical Co. Ltd., Tokyo, Japan) at one center, a chemiluminescent immunoassay kit (Immulite 2000 ACTH; Siemens Healthineers) at one center, and an enzyme immunoassay kit (E-test TOSOH II; TOSOH) at one center. The reference ranges of these kits were 6.24–18.0, 5.27–22.45, 5.0–25.0, 5.1–17.0, and 6.7–22.6 µg/dL, respectively. Serum DHEA-S concentrations were evaluated using a chemiluminescent immunoassay kit (Access DHEA-S; Beckman Coulter) at eight centers, a chemiluminescent immunoassay kit (Chemilumi DHEA-S; Siemens Healthineers) at one center, and a chemiluminescent immunoassay kit (Access DHEA-S; Siemens Healthineers) at one center. The ranges of measurement of these kits were 2.0–1,000, 15–1,000, and 3.0–1,500 µg/dL, respectively. The DHEA-S ranges differed depending on age and sex.

Supplementary Table 1 Frequency of common symptoms, signs, and comorbidities in Cushing syndrome compared with previous reports

	Frequency (%)			*P*-value		Frequency (%)	*P*-value		Frequency (%)	*P*-value	
**Symptom, sign, or comorbidity**	**Western report**[8]	**Japanese report**[16]	**Our study**	**vs. Western report**	**vs. Japanese report**	**Earlier group**	**vs. Western report**	**vs. Japanese report**	**Later group**	**vs. Western report**	**vs. Japanese report**
Menstrual disturbances	0.84	0.600	0.253	<0.001	<0.001	0.270	<0.001	<0.001	0.238	<0.001	<0.001
Easy bruising	0.62	0.438	0.356	<0.001	0.059	0.314	<0.001	0.048	0.400	0.001	0.347
Muscle weakness	0.56	0.515	0.208	<0.001	<0.001	0.196	<0.001	<0.001	0.220	<0.001	<0.001
Facial plethora	0.94	N/A	0.248	<0.001	N/A	0.196	<0.001	N/A	0.300	<0.001	N/A
Moon face	0.88	0.816	0.842	0.150	0.303	0.804	0.079	0.468	0.880	0.565	0.162
Striae	0.56	0.508	0.208	<0.001	<0.001	0.275	<0.001	0.001	0.140	<0.001	<0.001
Obesity	0.97	N/A	0.406	<0.001	N/A	0.479	<0.001	N/A	0.333	<0.001	N/A
Central obesity	N/A	0.807	0.594	N/A	<0.001	0.627	N/A	0.002	0.560	N/A	<0.001
Hypertension	0.74	0.883	0.792	0.139	0.006	0.824	0.112	0.136	0.760	0.446	0.011
Dyslipidemia	0.71	N/A	0.616	0.028	N/A	0.640	0.174	N/A	0.592	0.051	N/A
Depression	0.62	0.152	0.129	<0.001	0.313	0.137	<0.001	0.479	0.120	<0.001	0.347
Diabetes mellitus	0.50	0.452	0.327	0.001	0.007	0.333	0.024	0.058	0.320	0.015	0.040
Osteoporosis	0.50	0.362	0.467	0.598	0.027	0.405	0.280	0.334	0.521	0.885	0.018

Comparisons between rates in the previous studies from Western countries[8)] and those from Japan[16)] were carried out using one sample binomial test.

Patients were categorized into two groups based on their diagnosis date: within 5 years of the most recent case (Dec 2011–Nov 2016, later group) or earlier (Aug 2005–Nov 2011, earlier group)

N/A, not available
